# *Cryptosporidium hominis* gene catalog: a resource for the selection of novel *Cryptosporidium* vaccine candidates

**DOI:** 10.1093/database/baw137

**Published:** 2016-10-19

**Authors:** Olukemi O. Ifeonu, Raphael Simon, Sharon M. Tennant, Abhineet S. Sheoran, Maria C. Daly, Victor Felix, Jessica C. Kissinger, Giovanni Widmer, Myron M. Levine, Saul Tzipori, Joana C. Silva

**Affiliations:** 1 Institute for Genome Sciences, University of Maryland School of Medicine, 801 West Baltimore Street, Baltimore, MD 21201, USA; 2 School of Systems Biology, George Mason University, 10900 University Boulevard, Manassas, VA 20110, USA; 3 Center for Vaccine Development, Institute for Global Health, and Department of Medicine, University of Maryland School of Medicine, 685 West Baltimore Street, Baltimore, MD 21201, USA; 4 Department of Infectious Disease and Global Health, Tufts University Cummings School of Veterinary Medicine, 200 Westboro Road, North Grafton, MA 01536, USA; 5 Department of Genetics, Institute of Bioinformatics and Center for Topical and Emerging Global Diseases, University of Georgia, 500 D.W. Brooks Drive, Athens, GA 30602, USA and; 6 Department of Microbiology and Immunology, University of Maryland School of Medicine, 685 West Baltimore Street, Baltimore, MD 21201, USA

## Abstract

Human cryptosporidiosis, caused primarily by *Cryptosporidium hominis* and a subset of *Cryptosporidium parvum*, is a major cause of moderate-to-severe diarrhea in children under 5 years of age in developing countries and can lead to nutritional stunting and death. Cryptosporidiosis is particularly severe and potentially lethal in immunocompromised hosts. Biological and technical challenges have impeded traditional vaccinology approaches to identify novel targets for the development of vaccines against *C. hominis*, the predominant species associated with human disease. We deemed that the existence of genomic resources for multiple species in the genus, including a much-improved genome assembly and annotation for *C. hominis*, makes a reverse vaccinology approach feasible. To this end, we sought to generate a searchable online resource, termed *C. hominis* gene catalog, which registers all *C. hominis* genes and their properties relevant for the identification and prioritization of candidate vaccine antigens, including physical attributes, properties related to antigenic potential and expression data. Using bioinformatic approaches, we identified ∼400 *C. hominis* genes containing properties typical of surface-exposed antigens, such as predicted glycosylphosphatidylinositol (GPI)-anchor motifs, multiple transmembrane motifs and/or signal peptides targeting the encoded protein to the secretory pathway. This set can be narrowed further, e.g. by focusing on potential GPI-anchored proteins lacking homologs in the human genome, but with homologs in the other *Cryptosporidium* species for which genomic data are available, and with low amino acid polymorphism. Additional selection criteria related to recombinant expression and purification include minimizing predicted post-translation modifications and potential disulfide bonds. Forty proteins satisfying these criteria were selected from 3745 proteins in the updated *C. hominis* annotation. The immunogenic potential of a few of these is currently being tested.

**Database URL:** http://cryptogc.igs.umaryland.edu

## Introduction

Although young child mortality has dropped impressively since the millennium, almost six million deaths still occur annually in developing countries, with diarrheal diseases remaining the second most common cause of death after pneumonia (1). The Global Enteric Multicenter Study (GEMS), an enormous case-control study that investigated the burden, etiology and consequences of moderate-to-serve diarrhea (MSD) in children < 5 years of age in four sites in sub-Saharan Africa and three in South Asia (global regions where collectively 80% of young child diarrhea deaths occur) incriminated *Cryptosporidium* as one of the four predominant pathogens overall associated with MSD and as the second most common pathogen during the first 2 years of life, after rotavirus (2). GEMS also found that *Cryptosporidium* MSD was associated with linear growth stunting the ∼60 days following the acute MSD episode and increased by 8.5-fold the risk of death over the ∼60-day follow-up compared with matched control children. Although *Cryptosporidium*, a chlorine-resistant pathogen, also occurs in association with sporadic and outbreak water-related transmission in industrialized countries, it is to address the burden of disease in developing countries that there have been calls to undertake vaccine development efforts.

Two main species of the apicomplexan genus *Cryptosporidium* are associated with human disease. GEMS revealed that 80% of *Cryptosporidium* associated with cases were human-restricted *Cryptosporidium* *hominis*, while the *Cryptosporidium* *parvum* strains were also mainly anthroponotic genotypes. The majority of human infections in non-GEMS developing countries is attributed to *C. hominis* and, to a lesser degree, *C. parvum* (3–6). Other *Cryptosporidium* species are found in all vertebrate groups, with a few occasionally isolated from humans with diarrhea (3).

Vaccination remains one of the most successful and cost-effective methods of preventing the occurrence and spread of serious infectious diseases. The fact that only one parasitic vaccine has been licensed for human use (Mosquirix against *Plasmodium falciparum* malaria, approved only in 2015, for use in targeted groups) reflects the challenges associated with the design and development of effective anti-protozoal vaccines. Among the factors limiting the understanding of *C.* *hominis* biology and the development of anti-cryptosporidial vaccines has been the lack of a robust axenic *in vitro* culture system (7), although successful *in vitro* cultivation of *C. parvum* has recently been demonstrated (8).

Reverse vaccinology takes advantage of annotated pathogen genomes to identify genes encoding proteins with properties predicted to induce a host immune response against the pathogen. This approach permits the rational selection of vaccine components which can be subsequently validated experimentally to determine if they elicit immune responses and confer protection (9–11). The reverse vaccinology approach was first used to successfully identify the four components of the *Neisseria meningitidis* B vaccine (Bexsero) (12–14), wherein the genome sequence of a virulent isolate (MC58) was used to predict candidate surface-exposed or exported proteins. Following a similar approach, Maione et al. (15) identified four potential vaccine antigens against Group B streptococcus and demonstrated that a multivalent vaccine formulation using these antigens can confer broad serotype-independent protection. Reverse vaccinology is also being applied to other pathogens for which not licensed vaccines or other mature candidates exist, including *Porphyromonas gingivalis* and *Chlamydia pneumoniae* (16). The reverse vaccinology approach is particularly promising for organisms that, like *Cryptosporidium*, are difficult to maintain under routine laboratory conditions (13, 15, 17, 18).

**Figure 1.**
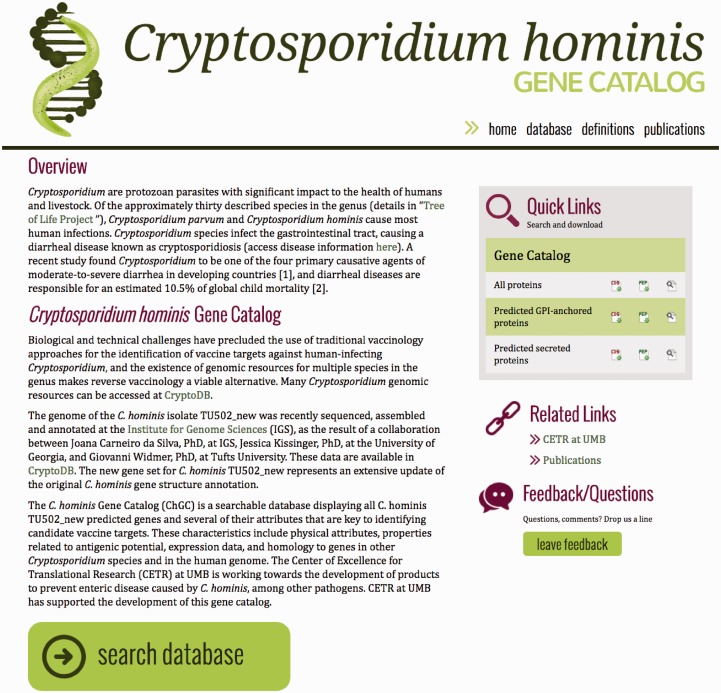
*Cryptosporidium hominis* gene catalog (ChGC). The landing page includes an overview of ChGC and links to related information and resources. Several data subsets are readily available for download (right hand bar), and the full dataset can be further queried with user-selected criteria (bottom button). Direct links to the definition of each criterion, as well as related publications, are also available (top right).

Advances in sequencing technologies and genome assembly and annotation methodologies have facilitated the generation of genomics resources for multiple species of *Cryptosporidium* (19). *Cryptosporidium* *parvum* (isolate IOWA II) was the first species with a published genome (20). The genome was found to be 9.1 Mbp in length, and its eight chromosomes assembled into 13 supercontigs, containing 3807 predicted protein-coding genes with an average length of 1795 base pairs (bp). At about the same time the genome of *C. hominis* (isolate TU502) was published (21). It was sequenced to a much lower depth of coverage because of limitations of biological material and technology available at the time. For example, the lack of conventional animal models to propagate this species limited the amount of DNA that could be generated for sequencing. Consequently, this assembly is comparatively more fragmented, with the likely eight chromosomes split among 1413 contigs, grouped into ∼240 scaffolds. Recently, we generated a much-improved annotated genome assembly for *C. hominis*, isolate TU502_2012 (22). Herein, we report a comprehensive functional annotation, and targeted manual structural validation, of this new *C. hominis* TU502_2012 gene set, with a view to generate a complete list of genes predicted to potentially be sporozoite, and most likely merozoite, surface-expressed. In addition, we developed a searchable online catalog of all *C. hominis* genes and their characteristics of interest in the context of vaccine development, including physical attributes, properties related to antigenic potential and expression data (Figure 1). As an example of this approach, we identified a multitude of proteins that could be evaluated as protective immunogens.

## Materials and Methods

### Genomic and transcriptomic data

This study relied on the use of the following genomics data:

- *Cryptosporidium hominis* TU502: whole genome sequence data (AAEL00 00 00 00); assembly and annotation (GCA_000006425.1) (21)
- *Cryptosporidium hominis* TU502_2012: whole genome sequence data (JIBM 00000000); assembly (submitted; requested public release); RNASeq data (SRX481527)
- *Cryptosporidium hominis* UKH1: whole genome sequence data (JIBN0 000 0000); assembly (submitted; requested public release);
- *Cryptosporidium parvum* Iowa II: whole genome sequence data (AAEE01 000000); assembly and annotation (GCA_000165345.1) (20). Note: this genome was recently re-annotated (23) but at the time of this study the updated annotation was not publicly available. Thus, all references to *C. parvum* Iowa II are based on the original annotation.
- *Cryptosporidium baileyi* TAMU-09Q1: whole genome sequence data (JIBL00 000000); assembly (submitted; requested public release);
- *Cryptosporidium meleagridis* UKMEL1: whole genome sequence data (JIBK00 000000); assembly (submitted; requested public release);
- *Cryptosporidium muris* RN66: whole genome sequence data (AAZY02000000); assembly and annotation (GCA_000006515.1);
- *Homo sapiens*: year 2014 (GRCh38.p1); assembly and annotation (GCA_000001405.16) (24).

The first version of the annotation of the genomes of *C. hominis* TU502_2012, *C. hominis* UKH1, *C.* *baileyi* TAMU-09Q1 and *C.* *meleagridis* UKMEL1 will be released soon (22). 

### Functional annotation

The structural and functional attributes of the 3745 protein-coding genes in the updated *C. hominis* assembly were identified using a variety of approaches. These include BlastP (25) searches against the proteome of other Apicomplexa, using the weight matrix BLOSUM62 and an *E*-value cutoff of 1e^−^^5^, HMMer version 3.0 (26) searches against the PFAM and TIGRfam databases of functional protein domains (27) and searches against the InterPro (28) and CDD (29) databases. Results from these analyses were then parsed using a custom script to assign product names, gene symbols, enzyme commission numbers and Gene Ontology terms, where available.

### Characterization of surface-expressed or secreted proteins and epitope identification

The targets of protective antibodies on microbial pathogens are typically associated with the surface of the pathogen or the infected host cell. Accordingly, TargetP (30, 31) was used to identify proteins predicted to be targeted to the secretory pathway with high reliability (reliability Classes 1 or 2). Proteins were predicted to be glycosylphosphatidylinositol (GPI)-anchored using GPI-SOM (32), PredGPI (33) and FragAnchor (34). The presence of five or more transmembrane helices is a strong indicator of a transmembrane protein; the presence of these transmembrane motifs was determined with TMHMM (35, 36). Prediction of antigens that may constitute robust immunogens was done by analysis of potential Major Histocompatibility Complex (MHC) Class I and MHC Class II epitopes with NetMHCpan and NetMHCIIpan, respectively (37–39).

### Manual curation of gene structure

Gene structure was manually validated for all genes predicted to be secreted or membrane-associated (determined by the presence of predicted GPI anchors or of at least five transmembrane motifs). The manually curated gene structural components included the location of the methionine start codon and the location of all intron–exon boundaries. The following data was used as evidence: *C. hominis* strand-specific RNAseq data generated from the oocyst stage (GenBank: SRX481527), ‘TopHat junctions’ [the set of reads predicted by TopHat (40) to span introns], homologous proteins from other *Cryptosporidium* species aligned against the *C. hominis* assembly using GMAP (41) and CEGMA proteins, a set of highly conserved eukaryotic genes (42). Manual validation consisted of visual inspection of each gene model, comparison against all available evidence and editing when necessary to conform to that evidence. Web Apollo (43) was used to visualize all evidence tracks and to modify gene models as necessary.

### Protein physical attributes

The proteins were characterized according to several physical properties, including predicted isoelectric point (44), molecular weight (44), numbers of cysteine residues (assumed to reflect potential disulfide bonds) or of potential glycosylation sites. We predicted two types of glycosylation sites, O-glycosylation and N-glycosylation sites, by use of the software NetNGlyc, NetOGlyc and GlycoEP (45–47). 

### Homology searches

*C. parvum* and human homologs were identified by running a BlastP search of *C. hominis* TU502_2012 proteins against the proteomes of *C. parvum* Iowa II (20) and human (48), respectively, with parameter values as described earlier. The presence of homologs of genes of interest was also determined in four other *Cryptosporidium* genomes, namely, *C. parvum* Iowa II*, C. baileyi* TAMU-09Q1*, C. meleagridis* UKMEL1 *and C. muris* RN66. We computed homology clusters of *Cryptosporidium* proteins using the pipeline described by Crabtree and collaborators (49), and used the Sybil comparative platform (49) to visualize and analyse the results.

### Identification of SNPs and small insertions/deletions (indels)

Sequence variants, in particular single nucleotide polymorphisms (SNPs) and small indels in *C. hominis* were identified based on the comparison of two strains: *C. hominis* TU502_2012 and *C. hominis* UKH1. In this case, the sequence reads of *C. hominis* UKH1 (SUB482088) were aligned to the new assembly of *C. hominis*, ChTU502_2012, using BWA (50). Sequence data was formatted using SAM tools (51) and Picard tools v.1.79 (http://broadinstitute.github.io/picard), and SNP variant calling and filtering using the Genome Analysis Toolkit GATK v2.2.5 (52). Identified variants were filtered according to the following parameter values: (DP < 12) ‖ (QUAL < 50) ‖ (SB > −0.10) ‖ {MQ0 ≥ 2 && [MQ0/(1.0 × DP)] > 0.1}. SNPs that passed the filter were attributed to non-coding or coding regions using VCFannotator (http://sourceforge.net/projects/vcfannotator) using as reference the annotation of ChTU502_2012.

### Expression dataset

Given the lack of *C. hominis* sporozoite RNAseq data, we used transcriptomic data from *C. parvum*. From CryptoDB (19), we extracted expression data representing transcriptomes of freshly excysted *C. parvum* sporozoites, as well as data for parasites collected 48- and 96-h post-infection in HCT-8 cells. These data were generated using SOLiD, paired end, strand-specific RNA sequencing (Hehl AB et al., unpublished data). In addition, we utilized amino acid data representing excysted sporozoite proteomes. These data originated from solubilized protein preparations analysed by 2D electrophoresis LC-MS/MS (53).

## Results

### Generation of a comprehensive set of putative antigens

**Figure 2.**
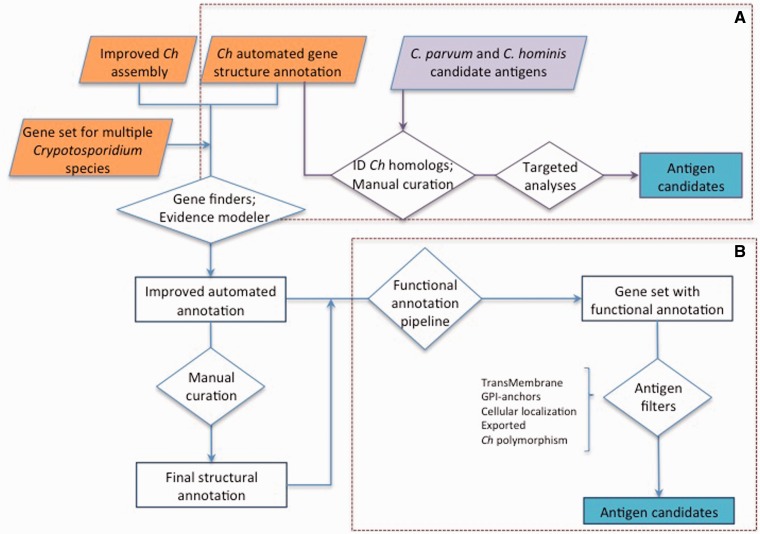
Approaches used for antigen identification. (**A**) Genes homologous to previously proposed *C. hominis* (*Ch*) or *C. parvum* antigens (purple) were identified among the gene set from the new *C. hominis* TU502_2012 genome assembly. The structural annotation of these genes was then manually curated, and targeted analyses were conducted to identify genes encoding proteins with the desired properties. (**B**) The structural annotation of *C. hominis* TU502_2012 was improved using information from related species and several of gene finders. The resulting gene set was assigned functional annotation. This gene set was then screened from desired properties. The gene structure of antigen candidates was manually curated.

We recently completed the sequencing, assembly and annotation of the genome of *C. hominis* genome isolate TU502 from a DNA sample generated in 2012 at Tufts University, named *C. hominis* TU502_2012. The isolate is believed to be the same that was sequenced in 2004 (21), except for the fact that it has been maintained by serial propagation in pigs for an additional 8 years. This effort resulted in a much-improved draft genome assembly for *C. hominis*. The *C. hominis* TU502_2012 genome assembly, with 119 contigs, is much less fragmented than the 1413-contig 2004 assembly (21), with the largest contig now the length of a chromosome. In this more comprehensive genome assembly, the average length of protein-coding genes is 500 bp longer than in the original annotation (22). The additional gene length resulted in a 25% increase in the fraction of the genome that encodes for proteins (Table 1). Based on this new gene set, we identified potential vaccine proteins using two bioinformatic approaches (Figure 2). In one approach, candidate antigens in *C. hominis* or *C. parvum* were identified from the literature (54–66), and their homologs were identified in the new *C. hominis* annotation. In a complementary approach, we used the complete *C. hominis* gene set to identify novel candidate antigens. The structure of all genes identified through either approach was manually validated (see Materials and Methods).

**Table 1.** Summary of assembly and annotation statistics for *Cryptosporidium* species. The data for the newly generated *C. hominis* TU502_2012 isolate (bold) show a significantly improved assembly and gene structural annotation for this species.

Species	Isolate	Assembly length (bp)	No. contigs	Largest contig (bp)	No. protein-coding genes	Average gene length (bp)	% coding
*C. hominis*	TU502 (2004)	8 743 570	1413	90 444	3886	1360	60.4
***C. hominis***	**TU502_2012**	**9 107 739**	**119**	**1 270 815**	**3745**	**1847**	**75.9**
*C. parvum*	Iowa	9 103 320	13	1 278 458	3807	1795	75.3

### Identification of putative antigens by homology to ‘known’ antigens

The first approach we took was to manually curate the gene structure of all *C. hominis* TU502*_*2012 genes with homology to known or proposed surface antigens (Figure 2). Potential antigens were identified from the literature. Using reverse vaccinology strategies to analyse the *C. hominis* TU502 (2004) genome (21), Manque et al. (66) identified potential antigens by focusing on proteins associated with the parasite surface, including those possessing multiple transmembrane motifs, signal peptides, GPI signal anchors and similarities with known pathogenic factors. Other studies have identified *Cryptosporidium* virulence factors using immunological and molecular methods. These virulence factors are predicted to be involved in processes such as adhesion, excystation, locomotion, invasion, membrane integrity, fatty acid metabolism and stress protection (54). Finally, some *Cryptosporidium* antigens were identified through a text search for ‘antigen’ in the CryptoDB database (www.cryptodb.org) (19). A total of 302 potential antigens were identified from these references. Of these, 132 proteins (44%) were reported as secreted, 185 (61%) as containing five or more transmembrane domains and 74 (24%) as containing GPI-anchor motifs, with a few proteins possessing more than one of these attributes. We re-evaluated these assignments with new or improved methods and found that only 52 of the 74 genes are now predicted to have GPI-anchored domains. We manually curated the structure of all 302 genes in the new *C. hominis* genome assembly (Materials and Methods). In total, 94 of these genes needed to be corrected, resulting in more accurate gene structures than those published in 2004.

### Identification of novel vaccine candidates

Vaccines that elicit antibody-mediated immunity are based on secreted proteins, including toxins, and/or on highly expressed, surface-exposed or membrane-associated proteins (13, 15, 67). We sought to complement the gene set above by utilizing a variety of bioinformatics tools to identify additional genes encoding proteins with these properties, and which might have been missed in previous studies due to incorrect or missing gene models in the 2004 annotation properties (Figure 2B). Among the complete set of 3745 protein-coding genes from the improved semi-automated annotation of *C. hominis* (Table 1), we identified 105 new antigen candidates, 41 of which have five or more transmembrane domains, 37 with GPI-anchor motifs and 29 that are targeted to the secretory pathway. We confirmed that, relative to the original assembly, these 105 genes are either newly identified, genes with a considerably altered structure or genes newly predicted using new software. The structure of these 105 new candidates was manually curated as described earlier.

**Figure 3.**
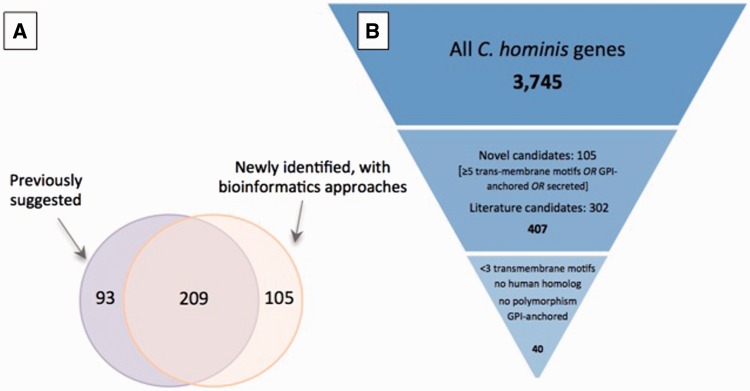
Selection of potential of *Cryptosporidium* vaccine candidates. (**A**) Overlap between set of potential antigens, one collected from the literature (purple) and the other generated using a bioinformatic screen for genes with predicted GPI-anchor motifs, secretion signals or at least five transmembrane motifs (orange). Of the total 407 potential antigens, roughly one-half were identified with both approaches. (**B**) Down-selection of genes to be used in immunogenicity experiments. The complete gene complement was first reduced by 90% to 407 candidates from (A), and a further 90% reduction resulted from the use of stricter criteria.

A total of 407 potential antigens were identified using at least one approach: 209 of the 302 previously identified putative antigens were also detected using our bioinformatic screen (Figure 3A); of the remaining 93 genes, approximately one-half have altered gene structures that may change the region containing signal peptides, which likely explains why they are no longer selected according to the criteria used in our screen.

### Rational selection of candidate vaccine proteins

The two combined approaches resulted in a set of 407 manually curated, potential antigens. To prioritize these genes, we characterized them according to relevant polymorphic and physicochemical properties. These properties include the possibility that the encoded protein will undergo post-translational modifications, suggestive of an intricate process of protein folding. In addition, we considered homology information, both across the *Cryptosporidium* genus and relative to the human proteome as cross-reactive antigens may produce undesired adverse effects upon vaccination.

Antigens often evolve rapidly, as a result of the selective pressure imposed by the host’s immune system (68, 69). Therefore, a relatively high rate of non-synonymous polymorphism and evidence of balancing selection have been used as criteria to identify new vaccine antigens (70, 71). However, evidence is now mounting that high rate of polymorphism in vaccine antigens contributes to vaccine evasion (72–74). To identify, and possibly eliminate, polymorphic loci from the pool of potential vaccine candidates, we estimated the number of SNPs between publicly available *C. hominis* isolates TU502_2014 and UKH1. A total of 230 protein-encoding genes have amino acid polymorphisms between these two isolates. In addition, we made use of publicly available gene expression data for *C. parvum*, to determine which genes are expressed during the sporozoite stage, since neutralizing antibodies are likely to target proteins expressed during this stage of development. Of the 3745 predicted protein-coding genes, 3597 are predicted to be expressed in the sporozoite stage, even though transcript abundance varies widely among genes.

Several additional selection filters were created based on homology information. All proteins with detectable homology to the human proteome were identified. In addition, we determined the taxonomic distribution of each *C. hominis* gene across the genus. These filters allow the elimination of potential antigens that may induce cross-reactions with human genes, and the rapid assessment of the potential taxonomic breadth of specific antigens.

Since proteins are often expressed in bacterial systems, the number and type of post-translational modifications are important considerations when choosing adequate vaccine candidates. Glycosylation is a type of post-translational modification resulting from the addition of N- and O-linked oligosaccharides to proteins. It assists in protein structural folding, transport and other functions (75, 76). Studies indicate that N-glycosylation of proteins is a rare event in apicomplexan parasites, even though it is an important post-translational modification in other eukaryotic phyla (77–81). For the full set of proteins, the median number of predicted N- and O-glycosylation sites per protein was 5 and 8, respectively, but both distributions were highly skewed, with maximum values ≥ 100. For the subset of 407 potential antigens, the median number of predicted N- and O-glycosylation sites per protein was 5 and 3, respectively. The median number of cysteine residues per protein, which can also be modified post-translation, was 7, with a maximum number of 227. For the subset of 407 selected genes, the median number of cysteine residues was nine per protein with a maximum number of 151. In most cases, the properties significant for the selection of candidate antigens have a higher rate of occurrence in the subset of 407 genes predicted to encode potential antigens compared with the full dataset (Table 2). Of these 407 genes, 33 were found to have amino acid polymorphism between the two *C. hominis* genomes and 216 had human homologs. Eliminating these, and further selecting genes with at most two predicted transmembrane motifs and genes predicted to be GPI-anchored, resulted in a list of 40 potential antigens, 39 of which have *C. parvum* homologs, that can be considered for further investigation as vaccine candidates (Figure 3). These can be further down-selected based on properties relevant for protein expression and with consideration of the chosen expression system, such as optimal isoelectric point for biochemical purification or optimal molecular weight for expression.

**Table 2.** Distribution of properties significant for the selection of candidate antigens in the full dataset and subset of candidate antigens

Desired properties	Full dataset (3745) (%)	Candidate antigens (407) (%)
Cellular localization: secreted	1	9
Predicted GPI-anchored	2	16
≥ 5 transmembrane motifs	6	56
≤ 6 cysteine residues	44	34
No. N-glycosylation sites^a^	11	9
No. O-glycosylation sites^a^	19	32
No. SNPs (strains TU502_2012 vs. UKH1)	94	92
No. human homolog	52	54
Conserved in *C. hominis*, *C. meleagridis, C. parvum*	60	65

a Using NetNGlyc, NetOGlyc, respectively.

### Cryptosporidium gene catalog

**Figure 4.**
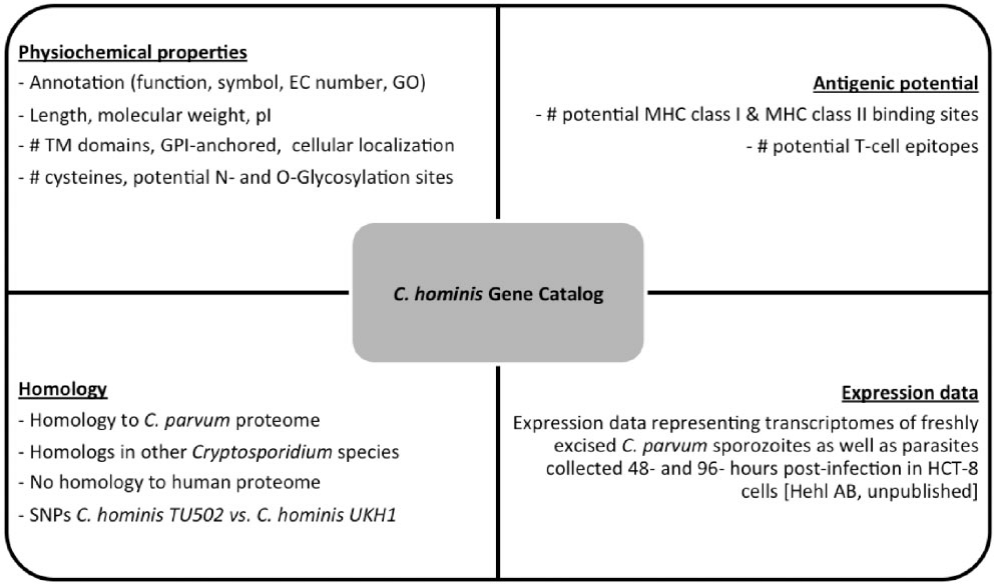
Properties stored in the C. hominis Gene Catalog (ChGC). The database contains a variety of searchable properties for each gene, including physicochemical properties, gene expression data, presence of potential T-cell epitopes and distribution of detectable homologs across the *Cryptosporidium* genus and in the human genome.

**Figure 5.**
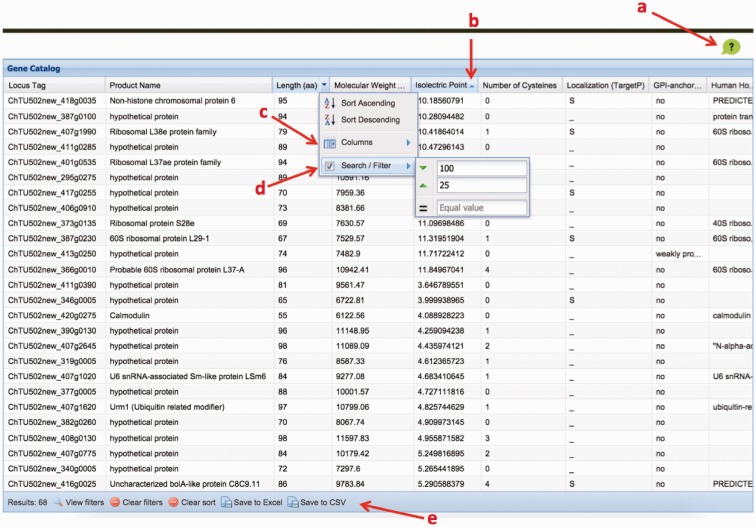
The ChGC interface. Key elements: (**a**) ‘Help’ button; (**b**) click on a column header to sort by that column; (**c**) ‘columns’ menu available in the drop-down menu on any column header is used to add hidden, or remove visible, columns; (**d**) ‘Sort/Filter’: multiple columns can be filtered to generate customized datasets of interest; (**e**) filtered datasets can be downloaded as an Excel or a CSV file, using these buttons.

We created a *C. hominis* gene catalog based on all the properties described earlier. The catalog is freely available online (http://cryptogc.igs.umaryland.edu). It contains all *C. hominis* genes and their characteristics, including physical attributes, properties related to antigenic potential and expression data (Figure 4). Users can sort or filter the genes based on each characteristic. For example, a query for proteins targeted to the secretory pathway, with no human homologs and at most 10 cysteine residues results in 14 hits (Figure 5). A quick query also shows that the estimated molecular weight for *C. hominis* proteins varies between 6.12 and 991.2 kDa, equivalent to 55–8756 amino acid residues.

Three sets of genes readily available for download, both in nucleotide and amino acid sequence fasta format include: all genes, genes that encode predicted GPI-anchored proteins or those whose products are predicted to be secreted. In addition, users can download the nucleotide and amino acid sequences of genes that meet specific user-defined criteria (Figure 5). The table of properties for all or a subset of filtered genes can also be downloaded in excel or comma separated values (CSV) format.

## Discussion

The GEMS (2) was designed to measure the burden, identify the major etiologic agents and assess the consequences of moderate-to-severe diarrhea (MSD) in children < age 5 years in the developing world. One conclusion of the study was the recognition that targeting the top 4–5 ranked diarrheal pathogens with effective interventions could reduce considerably the global morbidity and mortality burden of MSD.

Surprising to many was the finding that *Cryptosporidium* ranked second as the most important attributable pathogen associated with MSD in children below the age of 2 years. Whereas vaccines against the other three major pathogens either exist (rotavirus) or are undergoing clinical evaluation (enterotoxigenic *Escherichia* *coli* and shigellosis), efforts to develop a vaccine to protect humans against cryptosporidiosis have made little progress and no candidate has entered clinical trials. The advent of antiretroviral therapy and its widespread use in sub-Saharan Africa has markedly diminished the number of HIV-infected individuals that manifest overt immunodeficiency and as a result the frequency of cryptosporidiosis has in turn diminished along with interest and funding to combat this infection. GEMS’ revelation of the importance of *Cryptosporidium* has renewed interest in developing preventive as well as improved therapeutic measures to control in infants and toddlers in developing countries, including advocacy for developing vaccines. Given the practical obstacles associated with laboratory study of this parasite (7), reverse vaccinology is an attractive option to identify and prioritize antigens that may prove useful for the development of a well-tolerated and effective vaccine to prevent cryptosporidiosis.

With this in mind, our team has recently re-sequenced the TU502 isolate of *C. hominis*, assembled and annotated the genome, now designated TU502_2012 (22). The improved gene set, consisting of 3745 protein-coding genes, should provide the opportunity for new *in silico* analyses to identify potential immunogens. We are making this genomic database publicly available, with a view to stimulate additional investigators with expertise in reverse vaccinology to undertake research to develop *Cryptosporidium* vaccine candidates. Once *C. hominis* antigens of interest are identified, various vaccinology approaches can be adapted to assess their immunogenicity. Examples include assessment of the immune responses elicited in animal models or humans following immunization with protozoal antigens expressed in bacterial (82–84) or viral vectors (85–87), as virus-like particles (88, 89), as nanoparticles (90) or fused to carrier proteins, as has been done with *P. falciparum* and *Leishmania* proteins (82–90). Since *Cryptosporidium* is an intestinal protozoan, oral as well as parenteral routes of administration of the candidate vaccines should be studied, with and without adjuvants. Recent progress with a well-tolerated adjuvant for orally administered vaccines increases interest in a mucosal vaccine strategy (91).

Recently, genome sequences of additional isolates of *C. parvum* and *C. hominis* have become publicly available in CryptoDB (19). As annotation information for these genomes becomes available, a comparative analysis among *Cryptosporidium* species and isolates may help identify new antigens that will prove to have diagnostic value, since species identification currently entirely depends on cumbersome molecular genetic tools. The database may also help in the development of improved diagnostics of *Cryptosporidium* infection that may allow immunoassays that can identify the prevalent *Cryptosporidium* species in populations and geographic areas. Improved assays for species and sub-species differentiation can help elucidate the reservoirs of *Cryptosporidium*, likely modes of transmission and geographic spread, all of which can help formulate specific control measures.
